# Expression of the Matrix Metalloproteases 2, 14, 24, and 25 and Tissue Inhibitor 3 as Potential Molecular Markers in Advanced Human Gastric Cancer

**DOI:** 10.1155/2014/285906

**Published:** 2014-02-11

**Authors:** Sol de la Peña, Clara Luz Sampieri, Mariana Ochoa-Lara, Kenneth León-Córdoba, José María Remes-Troche

**Affiliations:** ^1^Biomedical Sciences Doctoral Program, Biomedical Research Center, University of Veracruz, 91190 Xalapa, Veracruz, Mexico; ^2^Institute of Public Health, University of Veracruz, 91190 Xalapa, Veracruz, Mexico; ^3^Public Health Master Program, University of Veracruz, 91190 Xalapa, Veracruz, Mexico; ^4^Dr. Miguel Dorantes Mesa Hospital, 91130 Xalapa, Veracruz, Mexico; ^5^Medical-Biological Research Institute, University of Veracruz, 91700 Veracruz, Veracruz, Mexico

## Abstract

*Background*. During progression of gastric cancer (GC), degradation of the extracellular matrix is mediated by the matrix metalloproteases (MMPs) and their tissue inhibitors (TIMPs): changes in the expression of these have been related to unfavorable prognosis in GC. *Objective*. To analyze the expression of certain MMPs and TIMPs in chronic superficial gastritis (SG) and GC. *Methods*. The expression of MMPs and TIMPs was determined using qRT-PCR; the expression was classified, using threshold cycle (*C*_*T*_) values, as very high (*C*_*T*_ ≤ 25), high (*C*_*T*_ = 26–30), moderate (*C*_*T*_ = 31–35), low (*C*_*T*_ = 36–39), or not detected (*C*_*T*_ = 40). Strength of association was estimated between the proteins, which were detected by Western blot, and the risk of developing GC. *Results*. We found a high expression of *MMP1*, *MMP2*, *MMP14*, *TIMP1,* and *TIMP3*; moderate one of *MMP9* and *MMP25,* and low one of *MMP13* and *MMP24* in both tissues. In absolute mRNA levels, significant differences were found in expression of *MMP2*, *MMP24,* and *MMP25*, which are overexpressed in GC compared with SG. The presence of the proteins MMP-14 and TIMP-3 was associated with the risk of developing GC. *Conclusions*. We consider that *MMP2*, *MMP24,* and *MMP25* and the proteins MMP-14 and TIMP-3 could be candidates for prognostic molecular markers in GC.

## 1. Background

Globally, gastric cancer (GC) represents the fourth most frequent neoplasia of the digestive system and is the second highest cause of mortality associated with malignant tumors [1, 2]. The elevated mortality rate of GC is probably due to the absence of specific symptoms in the early stages, which delays diagnosis of patients until the advanced stages when therapeutic options are limited or nil [2, 3].

Various models of carcinogenesis have been proposed for this type of tumor, but the most widely accepted is known as Correa's cascade [4, 5]. This model suggests that the gastric oncogenesis dynamic implies a multifactorial and sequential progression, apparently slow, of chronic superficial gastritis (SG) to premalignant lesions such as chronic atrophic gastritis, intestinal metaplasia, dysplasia, and finally GC [4, 5]. Likewise, the causal agent of SG has been identified as *Helicobacter pylori*, a bacteria designated as a type I carcinogen by the World Health Organization because its presence is associated with the development of GC [6, 7].

Currently, the main treatment for this type of tumor is surgical resection, which is effective in early stage tumors [8]. However, the prognosis for the patient with advanced GC is unfavorable; an average life expectancy of less than one year has been estimated in subjects with recurrent or unresectable tumors, even while undergoing chemotherapy [8, 9]. Other authors estimate a 5-year survival rate of 5 to 30% in these patients [3, 10]. Given this fact, it is necessary to identify reliable molecular markers that allow a proper diagnosis and predict the risk of recurrence and metastasis, as well as help to establish an accurate prognosis [9–12]. In this way, survival may improve through the provision of more effective medical and surgical treatment, monitoring of patients following surgery and during chemotherapy [8, 9], and an improved understanding of the biology of this disease facilitating the development of new therapeutic strategies [13].

Recent advances in the field of molecular biology, as well as the use of highly sensitive and specific technologies such as qRT-PCR [14], have enabled the identification of genes that express differentially in benign and malignant tissues, thereby revealing certain mechanisms involved in tumor progression [9, 10]. During tumor progression and metastasis, degradation of the extracellular matrix (ECM) occurs [15]; this process is mediated by the concerted action of various proteolytic enzymes, mainly the extracellular matrix metalloproteases (MMPs) [16, 17].

In humans, the family of MMPs comprises 24 zinc-dependent endopeptidases that degrade the components of the ECM and are secreted, in latent form (zymogen), by various cells such as fibroblasts, macrophages, neutrophils, and endothelial cells [16, 18]. For catalytic activation, these proteases require calcium as a cofactor, a neutral pH, and the proteolytic cleavage of their propeptides [16, 19]. Moreover, MMPs can modify or activate growth factors, tyrosine-kinase receptors, cytokines, chemokines, and cell-adhesion molecules, as well as other MMPs and proteases, thus participating in cellular signaling [16, 20].

Regulation of the expression and activity of MMPs is mainly controlled at the level of transcription, cellular compartmentalization, zymogen activation, and enzyme inhibition [21]. As the name implies, TIMPs are the main endogenous inhibitors of MMPs and this family comprises four members: TIMP-1, TIMP-2, TIMP-3, and TIMP-4, which bind reversibly to the catalytic site of the enzyme, forming a 1 : 1 stoichiometric complex and thereby inhibiting MMP activity [22].

Studies currently indicate that expression and activity in certain MMPs are enhanced in GC; this expression is associated with depth of invasion, lymph node metastasis, and unfavorable prognosis [23–25]. It has also been reported that repression of the expression of certain MMPs is associated with increased survival and favorable prognosis in GC patients [26–28]. It has therefore been suggested that MMPs be considered valuable potential candidates for molecular markers in GC [29].

In human GC, most recent reports have focused only on certain members of the family of MMPs and TIMPs; few studies have analyzed the full profile of the gene expression of these proteases and their inhibitors using highly sensitive and specific techniques such as qRT-PCR. To our knowledge, there is only one report that analyzes the gene expression of the entire family of MMPs and TIMPs in GC performed by qRT-PCR [11]. There are similarly few reports regarding the expression of these proteases in SG [30, 31]. In Mexico to date, there have been no studies concerning the genetic expression of the entire family of MMPs and TIMPs in GC and SG.

In this study, we examined the expression of certain MMPs and TIMPs in GC and SG, using qRT-PCR, which enables the identification of those MMPs that are over-expressed, repressed, or undetected during the progression of this disease. Moreover, possible associations are identified between the risk of developing GC and the presence of the protein, as detected by Western blot.

## 2. Methods

### 2.1. Patients

This study was approved by the Hospital Ethics Committee (Project: JE/035/07) and complies with the World Medical Association code of ethics (Declaration of Helsinki 1964, as revised in 2002). A case-control study was conducted, comprising patients with a confirmed histopathological diagnosis of GC as the cases, and those with a histopathological diagnosis of SG as the controls. These patients underwent upper digestive endoscopy between April 2007 and March 2012, authorizing their participation in this study by signing an informed consent. A total of 39 samples were included; 17 cases corresponded to advanced tumors (9 women and 8 men; average age: 58 ± 12.4 years; range 29–78 years) and 22 samples of SG, used as controls (9 women and 13 men; average age: 58.1 ± 15.8 years; range 24–100 years). None of the GC patients had received chemotherapy or radiotherapy prior to taking of a biopsy. Clinicopathological characteristics of the GC samples are shown in Table 1.

### 2.2. Extraction of mRNA

Clinical samples were collected in phosphate buffer solution and were kept in this solution less than 3 min, followed by immediate immersion in the tissue stabilization solution RNAlater (Ambion, Applied Biosystems, Foster City, CA, USA). The solution containing the tissue was kept overnight at 4°C and subsequently stored at −80°C. Tissue was homogenized in 1 mL of TRI reagent (Molecular Research Center, INC., Cincinnati, OH, USA). RNA extraction was performed following the instructions of the supplier until precipitation with 95% ethanol (Sigma-Aldrich, St. Louis, MO, USA). The solution was then placed in the column provided in the SV Total RNA isolation kit (Promega, Madison, WI, USA) and the instructions of the supplier were followed. Genomic DNA was digested using the DNase I enzyme supplied in the kit. Quantity and purity of the RNA were determined by measuring the optical density of each sample at 260 and 280 nm using a NanoDrop ND Spectrophotometer (Thermo Scientific, Wilmington, DE, USA).

### 2.3. Reverse Transcription

Total RNA (1 *μ*g) was incubated with random hexamers (Applied Biosystems) at 70°C for 10 min. The reverse-transcription reaction was then carried out for 1 hr at 42°C, using the High Capacity cDNA Reverse Transcription (Applied Biosystems) kit. The resulting cDNA was stored at −80°C.

### 2.4. Quantitative Real-Time Polymerase Chain Reaction

The qRT-PCR analysis was conducted using an Abi Prism 7500 Real Time PCR System (Applied Biosystems) following the conditions described by Nuttall and collaborators [32]. Primers and probes were designed and validated by Applied Biosystems for *18SRNA* (4308329), *MMP1* (Hs00899658_m1), *MMP2* (Hs00234422_m1), *MMP3* (Hs00968308_m1), *MMP8* (Hs01029057_m1), *MMP9 *(Hs00234579_m1), *MMP13* (Hs00233992_m1), *MMP14* (Hs00237119_m1), *MMP16* (Hs00254755_m1), *MMP24* (Hs00198580_m1), *MMP25* (Hs01554789_m1), *TIMP1* (Hs99999139_m1), and *TIMP3* (Hs00165949_m1). All standard curves were generated with 6 points for each of the genes MMPs and TIMPs and were prepared by performing serial dilutions from 20 ng of cDNA [32]; for *18SRNA *from 4 ng [32]. Amplification reactions were performed in triplicate. The number of PCR cycles at which amplification entered the exponential phase, known as the threshold cycle (*C*_*T*_), was determined and this number indicated the quantity of target RNA in each sample. *C*_*T*_ values were used to classify gene expression as very high (*C*_*T*_ ≤ 25), high (*C*_*T*_ = 26–30), moderate (*C*_*T*_ = 31–35), low (*C*_*T*_ = 36–39), or not detected (*C*_*T*_ = 40), following Nuttall and collaborators [32]. Absolute quantification of clinical samples was determined by comparison with the standard curve divided by the *18SRNA* *C*_*T*_ values (normalization factor).

### 2.5. Immunodetection of MMP-2, MMP-3, MMP-9, MMP-14, TIMP-1, and TIMP-3

Protein extraction was conducted using the organic phase obtained from the homogenized samples, following the protocol of the supplier (Molecular Research Center, INC.). Quantity of proteins was determined for each sample with the bicinchoninic acid assay (Sigma-Aldrich). Total protein equivalents (20 *μ*g) for each sample were mixed and boiled with sample buffer (50 mM Tris-HCl, pH 6.8, 10% glycerol, 2% SDS, 0.1% bromophenol blue, and 2% *β*-mercaptoethanol). The samples, molecular weight marker, and commercial standards for MMP-2 (Chemicon, Temecula, CA, USA), MMP-3 (Abcam, Inc., Cambridge, MA, USA), MMP-9 (R&D Systems, Minneapolis, MN, USA), MMP-14 (Chemicon), TIMP-1 (R&D Systems), and TIMP-3 (Abcam, Inc.) were electrophoresed on 10% polyacrylamide gels for the MMPs and 14% for the TIMPs and electrotransferred onto a separate polyvinylidene fluoride membrane (Bio-Rad, Hercules, CA, USA). The membranes were then blocked using a solution of 0.1% TBS-Tween 20 and low fat milk for 1 h at room temperature. Membranes were incubated with primary antibodies against *β*-actin, MMP-2, MMP-9 (polyclonal anti-rabbit, Cell Signaling Technology, Inc., Danvers, MA, USA), MMP-3, MMP-14, TIMP-1, and TIMP-3 (monoclonal anti-mouse, Millipore, Billerica, MA, USA). Each antibody was incubated in separate membranes (1 : 1000 dilution), following the instructions of the supplier. This was followed by blocking with antibody anti-rabbit IgG (Cell Signaling, Technology, Inc.) or anti-mouse IgG (Millipore), as appropriate, for 1 h at room temperature. Protein bands were visualized with chemiluminescence using the Amersham ECL Plus (GE Healthcare, Buckinghamshire, UK) system, as instructed by the supplier, followed by exposure to X-ray film.

### 2.6. Statistical Analysis

Standard curves were used to convert the *C*_*T*_ data to relative RNA levels and *C*_*T*_ values were expressed as an average ± standard deviation. To analyze differences in the expression of MMPs and TIMPs, between GC and SG, Mann-Whitney *U* tests were performed with the data normalized to *18SRNA*, and to determine the correlation between gene and protein expression, as well as the clinicopathological variables, the point-biserial correlation coefficient (*r*_pb_) was calculated. These analyses were conducted with the statistical software Sigma Stat (SPSS Inc., Chicago, USA), where a value of *P* < 0.05 was considered significant.

Strength of association was estimated between presence and absence of the proteins MMP-2 zymogen (72 kDa), MMP-2 active form (62 kDa), MMP-2 catalytic domain (45 kDa) [33], MMP-3 (54/59 kDa and 44/49 kDa), MMP-9/lipocalin (125 kDa), MMP-9 zymogen (92 kDa), MMP-9 active form (82 kDa), MMP-14 (60/66 kDa), TIMP-1/MMP-1 (66 kDa), TIMP-1 monomer (23/24 kDa), TIMP-3 dimer (50 kDa), and TIMP-3 monomer (24/33 kDa), with the risk of developing GC; likewise strength of association between the gene and protein expression and clinicopathological variables was measured by calculating the odds ratio (OR) and its 95% confidence interval (CI) with the statistical program EPIDAT 3.0 (Epidat Inc., PAHO, WA, USA).

## 3. Results

### 3.1. Expression of MMPs and TIMPs in Biopsies with GC and SG

**Figure 1:**
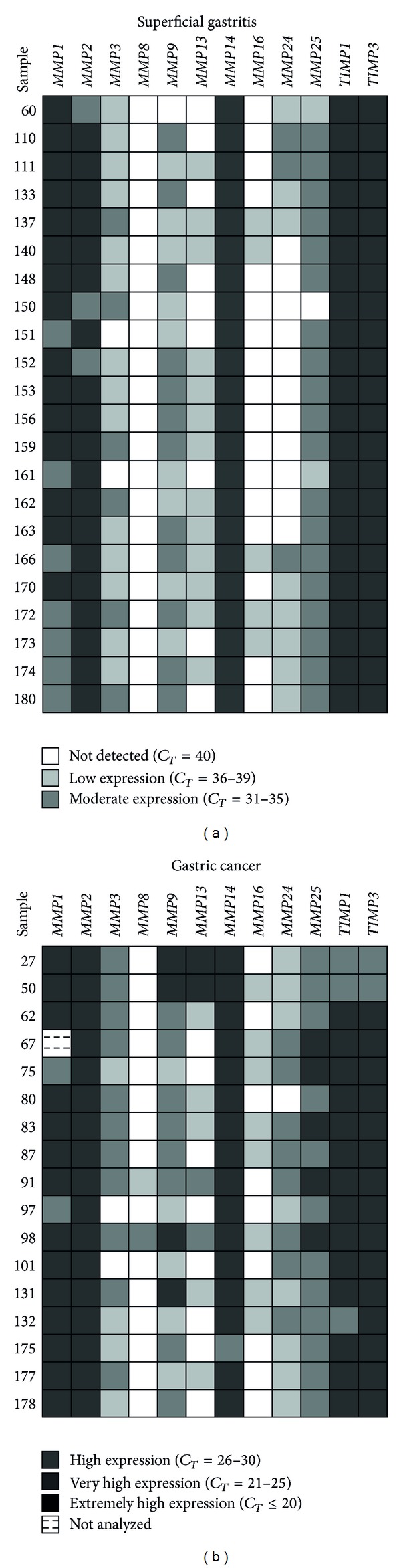
Genetic expression profile of certain MMPs and TIMPs determined by qRT-PCR in gastric cancer and superficial gastritis.

**Figure 2:**
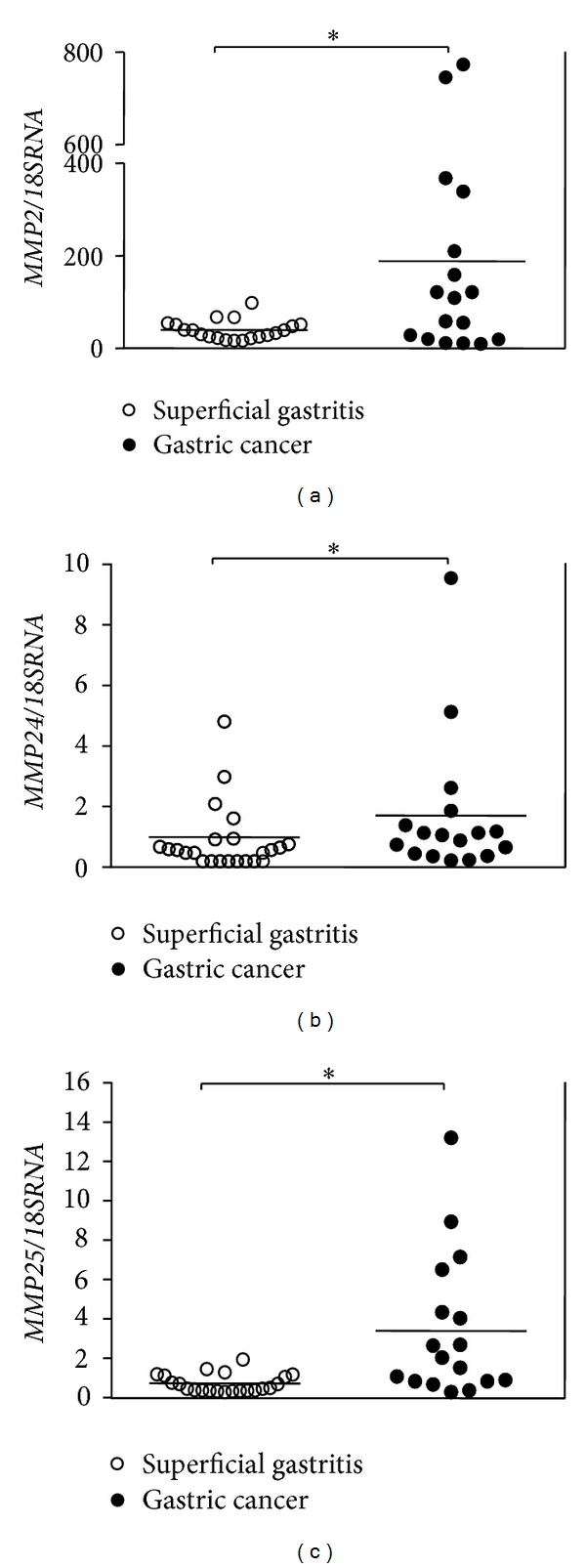
Absolute quantification, determined by qRT-PCR in gastric cancer and superficial gastritis. (a) *MMP2*, (b) *MMP24,* and (c) *MMP25*. Bars correspond to the mean. * Significant difference (*P* < 0.05).

The results indicate that expression of *MMP1* was mainly high (*C*_*T*_ = 26–30) in both tissues, except in 7/22 samples of SG and 2/17 of GC, which presented moderate expression (*C*_*T*_ = 31–35), and 1/17 samples of GC, which was not analyzed for this gene. For *MMP2*, high levels of expression were detected in 20/22 SG samples, while only 2/22 samples expressed moderately; in all GC samples, expression of *MMP2* was high (17/17). Likewise, expression of *MMP14*, *TIMP1,* and *TIMP3* was high in both tissues for all samples analyzed. *MMP9* expression was moderate in 12/22 SG samples, while 9/22 presented low levels (*C*_*T*_ = 36–39) and in 1/22, no expression of this protease was detected (*C*_*T*_ = 40); in GC, expression of *MMP9* was low (5/17), moderate (8/17), and high (4/17). In general, levels of *MMP25* were high for SG (19/22), except in 2/22 samples with low expression and 1/22 where no expression was detected; in GC, expression of this gene was moderate in 11/17 samples and high in only 6/17. In addition, the expression of *MMP3* was observed to fluctuate from low (13/22) to not detected (9/22) in the SG samples; expression in GC tended to be moderate in 11/17 samples; however, expression was low in 4/17 and not detected in 2/17 samples. Levels of expression of *MMP13* tended to be low or not detected in 9/22 and 13/22 SG samples, respectively; in GC, expression of this protease was not detected in 8/17 samples, while in the remainder, the observed levels were low (5/17), moderate (2/17), and high (2/17). In SG, levels of *MMP24* could vary since in 11/22 samples expression was not detected, while the rest of the samples presented low (8/22) and moderate expression (3/22); likewise, in GC, *MMP24* was expressed at moderate (8/17), low (8/17), and not detected (1/17) levels. For *MMP16*, expression was not detected in the majority of the SG samples (17/22), except in 5/22 samples which presented low levels of this transcript; in GC, this protease was expressed at low levels in 8/17 samples, while the other 9/17 samples presented no expression. In general, no expression of *MMP8* was detected in any SG samples, likewise in 15/17 samples of GC; the remaining 2/17 samples presented low and moderate levels of expression (Figure 1). Regarding absolute quantification of the transcripts, no significant differences were detected between GC and SG in terms of *MMP1*, *MMP3*, *MMP8*, *MMP9*, *MMP13*, *MMP14*, *MMP16*, *TIMP1,* and *TIMP3* expression. Conversely, significant differences were observed in the expression of *MMP2* (*P* = 0.043), *MMP24* (*P* < 0.001), and *MMP25* (*P* < 0.001), which were overexpressed in GC compared to SG (Figure 2). The median and interquartile range of the MMPs and TIMPs expression detected by qRT-PCR in GC and SG samples are shown in Table 2.

### 3.2. Analysis of MMP-2, MMP-3, MMP-9, MMP-14, TIMP-1, and TIMP-3 Expression Using Western Blot in GC and SG Samples

**Figure 3:**
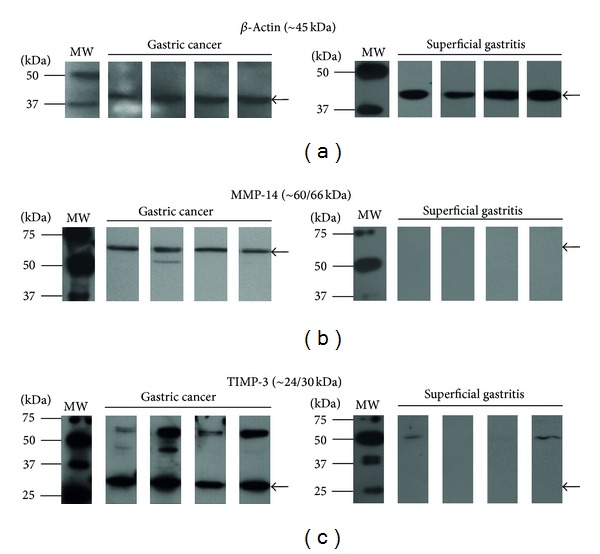
Immunodetection using Western blot in protein extracts of gastric biopsies with gastric cancer and superficial gastritis. (a) *β*-Actin, (b) MMP-14, and (c) TIMP-3.

The results indicate that proteins MMP-2 zymogen, MMP-2 active form, the catalytic domain of MMP-2 [33], MMP-3, MMP-9/lipocalin, MMP-9 zymogen, MMP-9 active form, TIMP-1/MMP-1, TIMP-1 monomer, and TIMP-3 dimer are not associated with the development of GC. Conversely, associations were detected between the risk of developing GC and MMP-14 (OR = 6.00, CI 95% = 1.02–35.27) and the TIMP-3 monomer (OR = 6.00, CI 95% = 1.02–35.27).  Figure 3, shows a representative example of the results obtained by the Western blot analysis for MMP-14 and TIMP-3. In addition, there was no association between protein expression and the clinicopathological variables of age, gender, size, and degree of differentiation.

### 3.3. Analysis of Correlation between Gene and Protein Expression

There was no correlation found between the gene expression of *MMP2*, *MMP3*, *MMP9*, *MMP14*, *TIMP1* and *TIMP3* and that of their proteins (*P* > 0.05). In addition, no correlations were detected between gene expression of *MMP1*, *MMP2*,* MMP3*, *MMP8*, *MMP9*, *MMP13*, *MMP14*, *MMP16*, *MMP24*, *MMP25, TIMP1 *and *TIMP3 *and the variables of age, gender, size and degree of differentiation (*P* > 0.05).

## 4. Discussion

This study showed the existence of differences in the expression of *MMP2*, *MMP24,* and *MMP25* between GC and SG, with expression significantly higher in GC compared to SG. With regard to *MMP24* and *MMP25*, to our knowledge, this is the second study to report overexpression of these proteases in gastric tumoral tissue. A previous study detected, through qRT-PCR, that *MMP24* and *MMP25* were overregulated by factors more than 4-fold in GC compared with peritumoral normal tissue [11]. In addition, there are no reports concerning the expression of *MMP24* in other types of tumors of the gastrointestinal tract. On the other hand, studies dealing with the expression of *MMP25* at mRNA or protein level are scarce in other types of gastrointestinal cancer. Increased expression of MMP-25, detected by immunohistochemical techniques, has only been reported in colorectal carcinoma samples compared to adjacent tissue [34]. For this reason, it would be of great interest to continue exploring *MMP24* and *MMP25* expression in both early and advanced stages of GC, since the possible function of these proteases during gastric oncogenesis is still unknown.

Baren and collaborators report no differences in *MMP2* expression between tumoral and normal peritumoral tissue [11], but these data are conflictive given that other authors have observed an increase of expression of *MMP2* mRNA and protein in gastric tumoral tissue compared to normal tissue [35] or tissue adjacent to the tumor [17, 36]. We consider the reason for the difference with the data of Baren and collaborators [11] is that these authors analyzed the expression of the entire family of MMPs in two types of tumors of the gastrointestinal tract (GC and esophageal cancer), reporting the results together without indicating to which tumor the expression corresponds. Elnemr and collaborators, using RT-PCR and Southern blot, detected expression of *MMP2* in 89/110 tumoral tissues compared to 23/110 control samples, associating expression of this protease with an unfavorable prognostic [17]. Our previous work reports significant differences in the expression of this protease between GC and normal tissue, but not between GC and SG [35]. Moreover, Allgayer and collaborators report a significant correlation has been detected between the staining intensity of MMP-2 with distant metastasis and with diffuse type GC (Lauren classification); however, there is no correlation with depth of tumoral infiltration (stage T), lymph node metastasis (stage N), infiltration of the blood vessels, Union for International Cancer Control (UICC) and Bormann classifications [37]. In contrast, Mönig and collaborators investigated possible correlations between the immunoreactivity of MMP-2 in tumoral cells with the current methods of classification, detecting that staining intensity was associated with stages T and N, distant metastasis (stage M), and the UICC classification [38]. On the other hand, Murray observed that 94% of the analyzed tumors showed positive staining for MMP-2, where 85% presented strong and 9% weak staining. This author also identified immunoreactivity of MMP-2 in macrophages [36]. Subsequently, Kabashima and collaborators did not find any correlation between the expression of MMP-2 and lymphatic invasion [39]. Additionally, the epithelial expression of MMP-2 has been associated in GC with male gender, advanced stage, advanced penetration depth, noncurative surgery, and an unfavorable prognosis, while stromal expression of MMP-2 has been related to advanced stage, diffuse type, and non-curative surgery [40]. Likewise, the positive expression of this protease has been reported as being associated with tumoral infiltration depth, lymph node metastasis, and the degree of differentiation of the tumor [25]. Equally, the immunoreactivity of MMP-2 is greater in gastric tumors with metastasis compared to primary gastric tumors [41, 42]. According to our findings and those reported by the authors cited previously, we consider that *MMP2* could be a potential candidate for use as a molecular marker for GC, although further study of both early and advanced tumors would be required in order to provide confirmation. In this way, it will be possible to determine differences in levels of expression of this protease during each one of the stages of this disease.

In this study, *MMP8 *was not detected in GC or in SG a finding which agrees with that of Baren and collaborators [11]. In addition, no differences were found in the expression of *MMP1*, *MMP3*, *MMP9*, *MMP13*, *MMP14*, *MMP16*, *TIMP1,* or *TIMP3*, despite the fact that several authors have reported the increased expression of these proteases and their inhibitors in GC, at both mRNA and protein levels [11, 17, 35, 39, 43]. Within this group of proteases, only *MMP9* [39], *MMP13 *[17], *MMP14* [43], and *TIMP1* [24] have correlated with depth of tumoral invasion, lymph node metastasis, and an unfavorable prognosis in GC patients.

In this work, the strength of association between the risk of developing GC and the presence of the proteins MMP-2, MMP-3, MMP-9, MMP-14, TIMP-1, and TIMP-3 was also evaluated. Our results indicate that only MMP-14 and the monomer of TIMP-3 are strongly associated with this risk. There is evidence of the participation of MMP-14 during gastric carcinogenesis, with reports of a strong intensity of staining of this protein in gastric tumoral tissue compared to healthy gastric mucosa [43]. Mori and collaborators observed negative staining of the stromal cells in the majority of the areas analyzed; however, they identified some positive cells, mainly in the areas adjacent to the edge of the cancerous cells [43]. Equally, elevated values of MMP-14 have been correlated with invasion, lymph node metastasis, and peritoneal dissemination [28, 43]. This protease has also been localized in the gastric tumors at the invasion front, mainly in the plasmatic membrane of the fibroblasts and cancer cells [28]. For this reason, it has been suggested that MMP-14 participates in the invasion of gastric tumors and could therefore be utilized as a molecular marker for this disease.

Regarding TIMP-3, few studies have explored this inhibitor at protein level; however, it has been detected using immunohistochemical techniques at low levels in diffuse type GC, as well as in poorly differentiated tumors and is associated with low survival [44]. Another report indicates that in tumors Bormann IV, expression of TIMP-3 is greater compared to tumors Bormann I, II or III [45]. In other types of gastrointestinal cancer, such as esophageal cancer (EC), expression of TIMP-3 has been found in the cytoplasm of basal, parabasal, and stromal cells of the normal tissue as well as in the cytoplasm of cancerous cells; however, it was observed that expression was reduced in the deep areas of the tumor in relation to the superficial areas [46]. In the same study, the authors found that TIMP-3 expression was correlated to the depth of tumoral invasion, lymph node metastasis, and infiltrative growth pattern and stage of the disease; thus, the prognosis for EC patients who present a reduction in expression of TIMP-3 is less favorable [46]. We consider TIMP-3 as a potential molecular marker for GC, although it is necessary to continue investigating the role of this inhibitor during all stages of gastric carcinogenesis.

Furthermore, our data indicate that there is no correlation between the gene expression of *MMP2*, *MMP3*, *MMP9*, *MMP14*, *TIMP1*, and *TIMP3* and that of their proteins; these results are consistent with Caenazzo and collaborators [47]. In contrast, other authors have reported a high expression of mRNA and the proteins of *MMP2* [17, 43] and *MMP14* [47] in GC. We consider that this discrepancy is due to variability in the specificity and sensitivity of the techniques used and to the existence of multiple levels of regulation in expression of MMPs and TIMPs.

In addition, there is no correlation or association between the expression of the genes (*MMP1*, *MMP2*,* MMP3*, *MMP8*, *MMP9*, *MMP13*, *MMP14*, *MMP16*, *MMP24*, *MMP25*, *TIMP1*, and *TIMP3*) and proteins (MMP-2 zymogen, MMP-2 active form, the catalytic domain of MMP-2 [33], MMP-3, MMP-9/lipocalin, MMP-9 zymogen, MMP-9 active form, TIMP-1/MMP-1, TIMP-1 monomer, TIMP-3 dimer, and TIMP-3 monomer) and the variables of age, gender, size, and degree of differentiation. This finding coincides with that of other authors [9, 25, 27, 43, 46, 48]; however, it has been reported that MMP-2 expression is correlated with male gender [38, 40].

## 5. Conclusions

In conclusion, this study represents the first partial pattern of gene expression of MMPs and TIMPs in GC and SG conducted in Mexican population and shows that the mRNA levels of *MMP2* are significantly higher in advanced GC compared to SG; interestingly, levels of *MMP24* and *MMP25* are also elevated in GC compared to SG. To our knowledge, this is the second report of differences in the expression of *MMP24* and *MMP25* in gastric tumors [11]. Moreover, we detected an association between the presence of MMP-14 and TIMP-3 and the risk of developing GC, for which reason we suggest these proteins to be molecular markers to distinguish patients with GC from those with SG. Likewise, we believe it is important to carry out more research to evaluate the clinical potential of *MMP2* in early stage GC patients and in those with SG since expression of *MMP2* has been considered a marker of distant metastasis in advanced tumors. Additionally, because of the paucity of information regarding *MMP24* and *MMP25*, more analysis is required to determine their function during the progression of GC; it is equally important to understand whether the expression of MMP-14 and TIMP-3 is involved with some activation mechanism of other MMPs, as has been reported previously for other members of the family of membrane-type MMPS and TIMPs. In addition, age, gender, size, and degree of differentiation present no correlation with the gene expression of *MMP1*, *MMP2*,* MMP3*, *MMP8*, *MMP9*, *MMP13*, *MMP14*, *MMP16*, *MMP24*, *MMP25, TIMP1, *and *TIMP3*. Neither is there association between these variables and the proteins zymogen, active and catalytic domain of MMP-2 [33]; MMP-3; zymogen, active and lipocalin-associated MMP-9; TIMP-1/MMP-1, TIMP-1 monomer, and the TIMP-3 monomer and dimer. We consider that expression of these proteases and their inhibitors at mRNA and protein level could represent a valuable instrument in the prognosis of this type of tumor, which is one of great public health concern.

## Figures and Tables

**Table 1:** Clinicopathological characteristics of patients with a diagnosis of gastric cancer included in the study.

Characteristic	*n* = 17 (%)
Age (years)	
Average ± standard deviation	58 ± 12.4
Range	29–78
Gender	
Feminine	9 (52.9)
Masculine	8 (47.1)
Histological type	
Adenocarcinoma	17 (100)
Histological differentiation	
Well	1 (5.9)
Moderate	2 (11.8)
Poor	11 (64.7)
Nondifferentiated	1 (5.9)
Unknown	2 (11.8)
Macroscopic classification	
Incipient	(0)
Advanced	17 (100)
Size	
Less than 20 mm	(0)
21–50 mm	8 (47.1)
Greater than 50 mm	9 (52.9)
Anatomic position (third)	
Upper	4 (23.5)
Middle	4 (23.5)
Lower	6 (35.3)
Upper/middle	1 (5.9)
Middle/lower	1 (5.9)
Total	1 (5.9)

**Table 2:** Median and interquartile range of MMPs and TIMPs expression in gastric cancer and superficial gastritis samples.

Gene	Gastric cancer			Superficial gastritis		
	Median (*C*_*T*_)	25%	75%	Median (*C*_*T*_)	25%	75%
*MMP1 *	29.50	27.20	28.51	29.00	25.92	27.86
*MMP2 *	27.97	27.20	28.51	27.05	25.92	27.86
*MMP3 *	36.71	35.10	37.32	34.54	32.79	37.85
*MMP8 *	40.00	39.98	40.00	40.00	40.00	40.00
*MMP9 *	35.15	34.01	35.96	34.45	31.15	35.84
*MMP13 *	39.25	38.73	40.00	39.25	35.87	40.00
*MMP14 *	28.35	28.08	28.35	27.66	26.93	28.09
*MMP16 *	39.66	39.52	39.90	40.00	38.58	40.00
*MMP24 *	38.90	36.16	40.00	35.74	34.33	37.22
*MMP25 *	33.68	32.84	34.37	31.69	29.97	32.25
*TIMP1 *	27.96	27.65	28.40	28.49	28.20	29.49
*TIMP3 *	28.07	27.27	28.75	27.47	26.12	29.06

*C*_*T*_: cycle threshold.
